# Zoonotic Pathogens in Ticks from Migratory Birds, Italy

**DOI:** 10.3201/eid2612.181686

**Published:** 2020-12

**Authors:** Elena Battisti, Katharina Urach, Adnan Hodžić, Leonida Fusani, Peter Hufnagl, Gerit Felsberger, Ezio Ferroglio, Georg Gerhard Duscher

**Affiliations:** Università degli Studi di Torino, Turin, Italy (E. Battisti, E. Ferroglio);; University of Veterinary Medicine Vienna, Vienna, Austria (K. Urach, A. Hodžić, L. Fusani, G.G. Duscher); University of Vienna, Vienna (K. Urach, L. Fusani);; Austrian Agency for Health and Food Safety, Vienna (P. Hufnagl, G. Felsberger, G.G. Duscher)

**Keywords:** migratory birds, zoonoses, Crimean-Congo hemorrhagic fever, Rickettsia, ticks, vector-borne infections, birds, Italy, viruses, bacteria

## Abstract

Migratory birds can transport infected ticks across continents. We evaluated pathogens in ticks collected from migratory birds in Italy. We found DNA from *Rickettsia aeschlimannii*, *R. africae*, and *R. raoultii* bacteria, all of which can cause disease in humans. Bird migrations might facilitate the spread of these pathogens into new areas.

Migratory birds can be biological and mechanical carriers of viruses, bacteria, and protozoa. They also can transport infected ectoparasites, such as ticks, across continents, enabling the spread of these vectors and their pathogens into new ecologic niches. Several studies have reported the *Borrelia burgdorferi* sensu lato, spotted fever group (SFG) rickettsiae, and Crimean-Congo hemorrhagic fever virus (CCHFV) in *Ixodes ricinus* and *Hyalomma marginatum* ticks collected from birds that migrate annually from Africa to Europe (*1*,*2*). The role of migratory birds as carriers of vectorborne pathogens in Italy is poorly understood. To assess the risk for introduction of zoonotic microbial agents in Europe by migratory birds, we investigated microorganisms in ticks collected from migratory birds in Italy.

## The Study

We conducted fieldwork activities at the Ponza Ringing Station on the island of Ponza (Central Tyrrhenian Sea, Italy; 40°55¢N, 12°58¢E) during spring (March–May) 2016 and 2017. We captured 744 migratory birds belonging to 20 different species (Table) during regular ringing procedures and checked them for ticks. Fourteen bird species were long-distance migrants that wintered in sub-Saharan Africa, and 6 were partial migrants, such as the blackbird (*Turdus merula*), the dunnock (*Prunella modularis*), the Eurasian blackcap (*Sylvia atricapilla*), the European robin (*Erithacus rubecula*), the song thrush (*Turdus philomenos*), and the subalpine warbler (*Sylvia cantillas*).

**Table:** Sampled bird species, ticks, and *Rickettsia* PCR positivity, Italy, 2016–2017*

Year, bird species	No. birds	Tick species found	No. pathogen-positive ticks/no. tested ticks	*Rickettsia*		
				*R. aeschlimanni*	*R. africae*	*R. raoultii*
2016						
Barn swallow (*Hirundo rustica*)	18	NA	NA			
Blackbird (*Turdus merula*)	1	*Ixodes ventalloi*	0/1			
Black redstart (*Phoenicuros ochruros*)	29	*I. ventalloi*	0/2			
		*Hyalomma* sp.	1/3	1	0	0
Eurasian blackcap (*Sylvia atricapilla*)	1	NA	NA			
European robin (*Erithacus rubecula*)	22	*I. frontalis*	0/1			
Garden warbler (*Sylvia borin*)	83	NA	NA			
Icterine warbler (*Hippolais icterina*)	19	*Hyalomma* sp.	1/2	0	1	0
Northern weathear (*Oenanthe oenanthe*)	1	*Hyalomma* sp.	1/1	1	0	0
Pied flycatcher (*Ficedula hypoleuca*)	21	*Hyalomma* sp.	1/5	1	0	0
Redstart (*Phoenicuros phoenicuro*)	14	*Hyalomma* sp.	7/9	7	0	0
Spotted flycatcher (*Muscicapa striata*)	25	NA	NA			
Subalpine warbler (*Sylvia cantillas*)	1	*I. frontalis*	0/1			
Tree pipit (*Anthus trivialis*)	1	*Hyalomma* sp.	1/2	1	0	0
Whinchat (*Saxicola rubetra*)	38	*I. frontalis*	0/1			
		*Hyalomma* sp.	4/13	4	0	0
Whitethroat (*Sylvia communis*)	92	*Ixodes* sp.	0/1			
		*Hyalomma* sp.	10/24	9	0	1
Willow warbler (*Phylloscopus trochilus*)	1	NA	NA			
Wood warbler (*Phylloscopus sibilatrix*)	1	*Hyalomma* sp.	0/3			
2017						
Barn swallow (*H.o rustica*)	20	*Hyalomma* sp.	0/1			
Black redstart (*P. ochruros*)	3	*H. rufipes*	1/1	0	1	0
Collared flycatcher (*Ficedula albicollis*)	1	*H. rufipes*	1/1	1	0	0
Dunnock (*Prunella modularis*)	1	*Hyalomma* sp.	0/1			
Eurasian blackcap (*S. atricapilla*)	48	NA	NA			
European robin (*E. rubecula*)	39	*H.rufipes*	2/10	2	0	0
Garden warbler (*S. borin*)	30	NA	NA			
Icterine warbler (*H. icterina*)	41	NA	NA			
Northern weathear (*O. oenanthe*)	1	*H. rufipes*	1/1	1		
Pied flycatcher (*F. hypoleuca*)	30	*Hyalomma* sp.	0/1			
Redstart (*P. phoenicuros*)	25	*H. rufipes*	8/12	8	0	0
Song thrush (*Turdus philomenos*)	4	*H. rufipes*	1/1	1	0	0
		*Hyalomma* sp.	0/3			
Spotted flycatcher (*M. striata*)	24	*H. rufipes*	1/2	1	0	0
Subalpine warbler (*S. cantillas*)	1	*Hyalomma* sp.	0/1			
Tree pipit (*A. trivialis*)	2	*Amblyomma marmoreum*	1/1	1	0	0
		*Hyalomma* sp.	0/1			
Whinchat (*S. rubetra*)	43	*H. rufipes*	5/7	5	0	0
Whitethroat (*S. communis*)	57	*H. rufipes*	2/5	2	0	0
Willow warbler (*P. trochilus*)	1	*Hyalomma* sp.	0/1			
Wood warbler (*P. sibilatrix*)	5	*H. rufipes*	1/1	1	0	0
		*Hyalomma* sp.	0/4			

*NA, no ticks collected.

We collected 231 engorged ticks and identified them using standard morphologic keys (*3*) and PCR amplification of the internal transcribed spacer (ITS) region when possible (*4*). We used commercial kits for RNA (High Pure Viral Nucleic Isolation Kit; Roche Diagnostics, https://diagnostics.roche.com) and DNA (High Pure PCR Template Preparation Kit; Roche Diagnostics) extraction. We used the RealStar CCHFV RT-PCR Kit 1.0 (Altona Diagnostics, https://www.altona-diagnostics.com) for CCHFV detection; we used conventional PCR with protocols described elsewhere (*5*) to detect DNA from *Babesia* spp., *Anaplasma* spp., *Ehrlichia* spp., SFG rickettsiae, and *Borrelia* spp. We used DNA from *Babesia canis* (dog 825/08, 1:10 diluted), *Anaplasma phagocytophilum* (cattle 2008/13, 1:10 diluted), *Ehrlichia canis* (clone), *Rickettsia raoultii* (clone) and *B. burgdorferi* (clone) as positive controls for each amplification.

Using PCR amplification of the ITS region, we identified 94 ticks at the species level: *H. marginatum* complex (5 larvae, 82 nymphs), *I. frontalis* (3 nymphs), *I. ventalloi* (3 nymphs), and *Amblyomma marmoreum* (1 nymph). Amplification of the ITS region failed in the remaining ticks, identifying only the genus; these ticks were mostly *Hyalomma* spp. (1 larva, 118 nymphs) or *Ixodes* spp. (3 larvae, 14 nymphs, and 1 adult).

Of the analyzed ticks, 50 tested positive for SFG rickettsiae DNA; the overall prevalence was 21.7% (95% CI 16.8%–27.4%). To determine the species, we amplified a fragment of the *ompA* gene in all the SFG rickettsiae–positive ticks (*5*). Positive amplicons were sequenced by LGC Genomics (https://www.lgcgroup.com) and compared with sequences deposited in GenBank. Results revealed *R. aeschlimannii* DNA in 47 (94.0% [95% CI 83.8%–97.9%]) of 50 ticks (Table). We identified 46 sequences identical to an *R. aeschlimanni* strain documented from Egypt (GenBank accession no. HQ335157), Turkey (GenBank accession no. MF379299), and Italy (GenBank accession no. MH532239) and 1 sequence identical to *R. aeschlimanni* strain RH (GenBank accession no. HM050286) from Senegal; the latter strain differed from the others by 1 nt (T instead of C at nt 425). Two (4.0% [95% CI 1.1%–13.5%]) sequences were identical to *R. africae* (GenBank accession no. HQ335132), and 1 (2.0% [95% CI 0.4%–10.5%]) sequence was identical to *R. raoultii* (GenBank accession no. MF166732). We also screened a subset of positive ticks using primers targeting a fragment of the *gltA* gene (*5*), confirming the results obtained with the *ompA* gene. No ticks tested positive for other microorganisms.

## Conclusions

Although ticks of the *H. marginatum* species complex (i.e., *H. marginatum*, *H. rufipes*, *H. turanicum*, and *H. isaaci*) are the most widespread ticks in Africa, they also have been found in some countries in Europe, such as the United Kingdom (*6*). These tick species are also vectors for CCHFV, which occurs mainly in Africa and southeastern Europe and can cause life-threatening disease in humans. *Hyalomma* ticks are vectors and reservoirs of this virus; birds, which are the primary hosts for the immature stages of these ticks, can maintain and spread the virus into new areas through migration (*7*).

*R. aeschlimannii* and *R. africae*, which are zoonotic bacterial species endemic to Africa, are transmitted by ticks belonging to the genera *Hyalomma* and *Amblyomma*. However, these bacteria have also been detected in ticks from other regions, such as Oceania, the Caribbean islands, and Europe (*7*–*9*). Autochthonous cases of human rickettsiosis caused by *R. aeschlimannii* have been recently described in Greece (*10*) and Italy (*11*). We detected *R. africae* in *H. rufipes* and *R. raoultii* in *Hyalomma* sp., which are not known vectors for these pathogens. Because we did not test the birds for *Rickettsia* spp. and the ticks were engorged, we cannot exclude the possibility that the ticks acquired these microorganisms by feeding on positive birds. Nevertheless, our results agree with those observed in a study in Italy (*12*) and confirm the circulation of these *Rickettsia* species into areas to which they are not endemic. They also highlight role of migratory birds in the passive transportation of infected ticks.

Although no ticks tested positive for CCHFV in our study, some studies report this virus in *H. marginatum* complex ticks attached to birds migrating from Africa to Europe (*13*). Migratory birds might have contributed to the establishment of the CCHFV in Spain (*14*). Moreover, climate change could cause prolonged, warmer, and drier summers and autumns. These seasonal changes might lead to the establishment of autochthonous populations of *Hyalomma* ticks in areas previously free of these vectors. Finally, RNA of another relevant human pathogen, the recently discovered Alkhurma hemorrhagic virus (*15*), has been detected in ticks of the *H. marginatum* complex.

In summary, we found zoonotic bacteria in ticks carried by birds across their migratory routes and assessed the risk for pathogen introduction in Italy. However, further studies are needed to clarify the role of these ticks in the epidemiology of zoonotic pathogens.
